# Reduction of hemagglutination induced by a SARS-CoV-2 spike protein fragment using an amyloid-binding benzothiazole amphiphile

**DOI:** 10.1038/s41598-024-59585-4

**Published:** 2024-05-29

**Authors:** Meihan Li, Sascha Castro Lingl, Jerry Yang

**Affiliations:** Department of Chemistry and Biochemistry, University of California, San Diego, 9500 Gilman Drive, La Jolla, 92093-0358 USA

**Keywords:** Drug discovery and development, Peptides, Protein aggregation

## Abstract

COVID-19 infection is associated with a variety of vascular occlusive morbidities. However, a comprehensive understanding of how this virus can induce vascular complications remains lacking. Here, we show that a peptide fragment of SARS-CoV-2 spike protein, S192 (sequence 192-211), is capable of forming amyloid-like aggregates that can induce agglutination of red blood cells, which was not observed with low- and non-aggregated S192 peptide. We subsequently screened eight amyloid-binding molecules and identified BAM1-EG_6_, a benzothiazole amphiphile, as a promising candidate capable of binding to aggregated S192 and partially inhibiting its agglutination activity. These results provide new insight into a potential molecular mechanism for the capability of spike protein metabolites to contribute to COVID-19-related blood complications and suggest a new therapeutic approach for combating microvascular morbidities in COVID-19 patients.

## Introduction

Coronavirus disease 2019 (COVID-19), caused by the severe acute respiratory syndrome coronavirus 2 (SARS-CoV-2) virus infection, is an ongoing global pandemic that has resulted in a significant impact on public health worldwide^1,2^. Symptoms of COVID-19 can range from mild to critical, with the most severe cases leading to pneumonia and acute respiratory distress syndrome (ARDS)^3^. Recently, there has been an increase in reported COVID-related cardiovascular complications and diverse coagulopathies^4–8^. Though the underlying pathogenesis of COVID-19 is not yet fully understood, the virulence of the pathogen is closely linked to its spike protein, a protruding membrane protein that plays a critical role in viral entry^1,9,10^.

SARS-CoV-2 spike protein is composed of 1273 amino acids and consists of two functional subunits: S1 for the host cell receptor binding and S2 for facilitating viral and cellular membrane fusion^9,10^. It has been reported that exposure to spike protein can induce the formation of abnormal dense clots, potentially causing blood hypercoagulation and impairing fibrinolysis^4,7,8^. Notably, these clots formed by spike protein subunit S1 were Thioflavin T (ThT)-positive, consistent with the presence of amyloid-like species^7^.

Amyloids are protein aggregates characterized by the ultrastructural fibrillar morphology, Congo red positivity and nucleation-dependent polymerization kinetics by Thioflavin T^11^. Peptides and proteins that accumulate and aggregate into amyloids are the hallmarks of neurodegenerative diseases, such as β-amyloid (Aβ) in Alzheimer’s and α-synuclein in Parkinson’s disease^12–14^. Studies have suggested that some amyloids, such as Aβ and human serum amyloid P component (SAP), are involved in agglutination and immune modulation, targeting different types of cells and pathogens^15,16^.

Recently, several 20-amino acid long segments within the SARS-CoV-2 spike protein were generated and reported to exhibit amyloidogenic properties^11^. Among them, S192 peptide, with the amino acid sequence 192-211 FVFKNIDGYFKIYSKHTPIN, was identified as the most amyloidogenic segment, fulfilling all three characteristics common to all amyloids^11^. Notably, this group found that full-length spike protein did not form amyloids on its own under similar conditions as the peptide segments. However, when the spike protein was incubated with neutrophil elastase (NE), a serine protease involved in immune responses, for a duration of 24 h in vitro, the formation of amyloid-like fibrils was observed. These fibrils exhibited clear branching, with the most amyloidogenic NE-induced fragments from the spike protein containing the S194 peptide sequence FKNIDGYFKI (spike protein amino acid residues 194-203)^11^.

**Figure 1:**
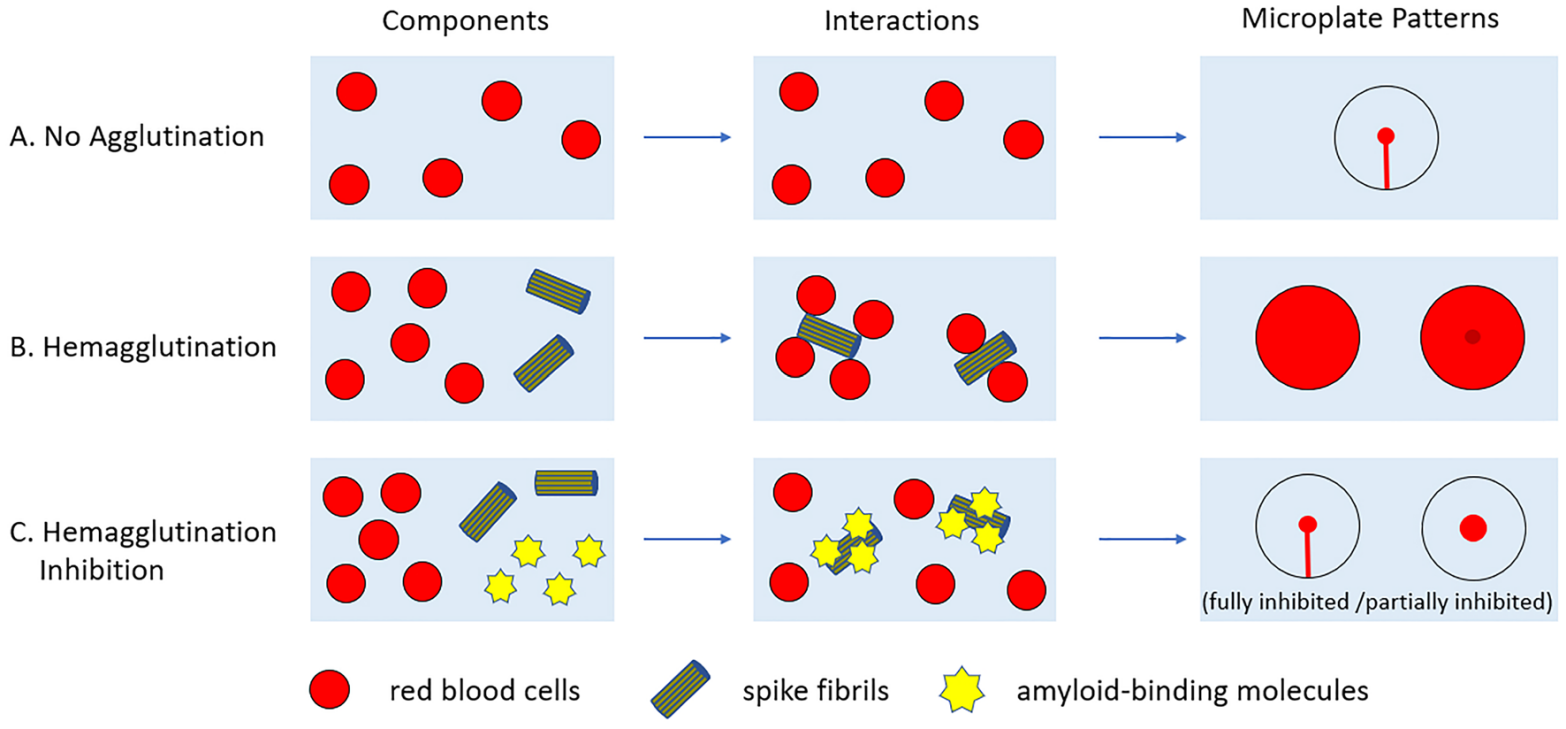
A schematic illustration of a microplate assay to examine hemagglutination induced by soluble/suspended amyloids and inhibition of hemagglutination by amyloid-targeting small molecules. (**A**) red blood cells with no agglutination, (**B**) amyloid fibrils generated from spike protein-derived segments (here, denoted simply as “spike fibrils”) induce agglutination of red blood cells, (**C**) amyloid-binding molecules form bio-resistive coatings on the spike fibrils, resulting in inhibition of the interaction between red blood cells and spike fibrils. The rightmost column illustrates the visual evidence for no agglutination, hemagglutination and full/partial inhibition of agglutination in a microplate assay.

Studies have shown that aggregated S192 and S194 participate in fibrin formation and fibrinolysis in in vitro experiments, suggesting a potential role in coagulation and hemagglutination-like activity^7,11^. Hemagglutination is the process where virus particles attach to red blood cells, causing them to clump together^17–19^. Although SARS-CoV-2 virus lacks the hemagglutinin protein typically responsible for inducing hemagglutination in influenza viruses, researchers have found that SARS-CoV-2 virus can still induce hemagglutination through the binding between N-glycans on the full-length SARS-CoV-2 spike protein and host cell glycoconjugates, such as sialic acids (SA)^5^. Here, we investigated whether the spike protein-derived segments S192 and S194, which do not naturally contain glycosylation sites, exhibit hemagglutination activity and show, for the first time, that only aggregated forms of S192 could induce agglutination of red blood cells (Fig. 1). We also demonstrate proof-of-concept that an amyloid-targeting small molecule could partially attenuate aggregated S192-induced hemagglutination.

## Results

**Figure 2:**
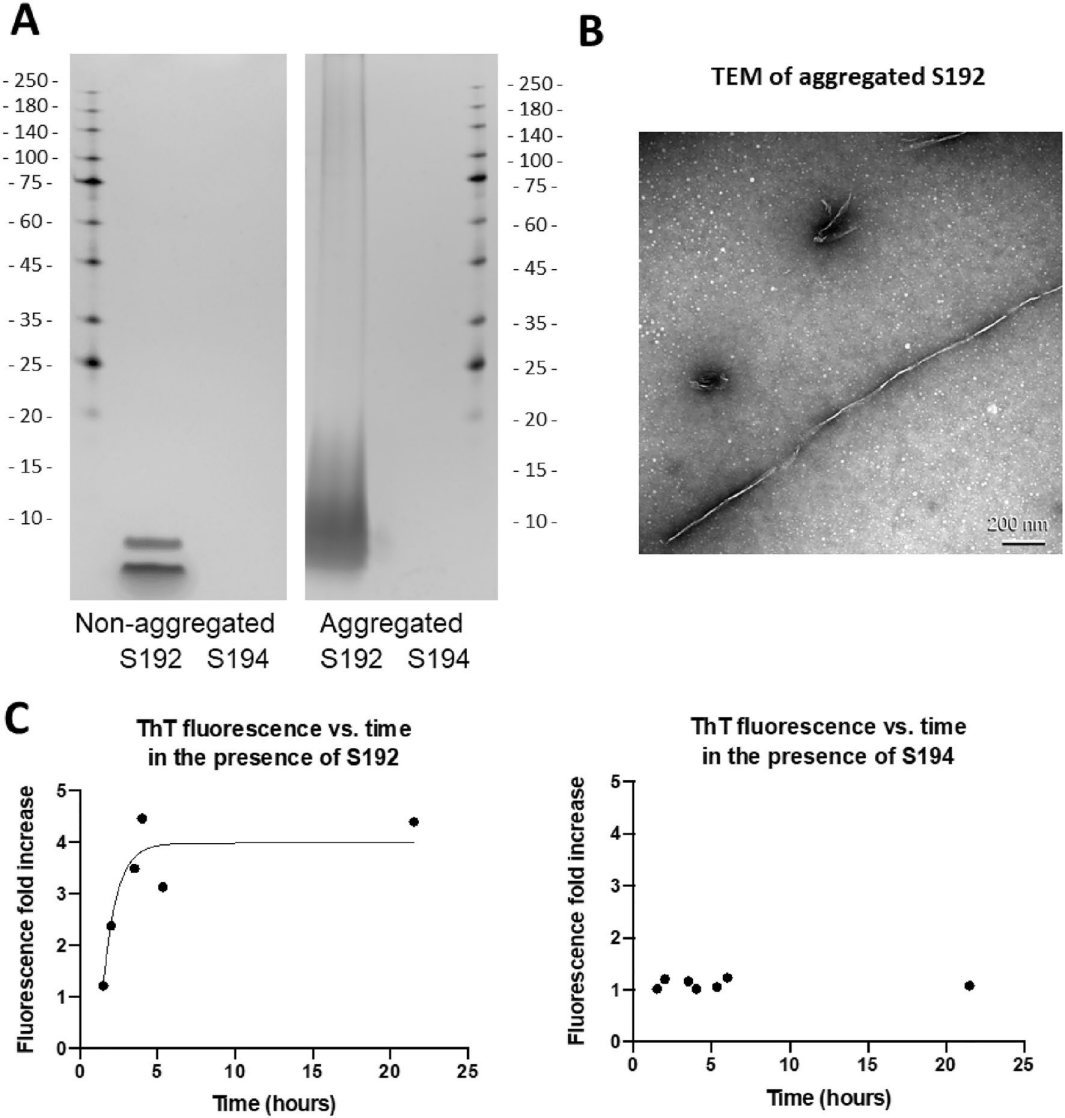
Characterization of SARS-CoV-2 spike segments, S192 and S194. (**A**) Silver stained SDS-PAGE gel of non-aggregated and aggregated spike segments. (**B**) Negatively stained TEM ultrastructure of aggregated S192. Scale bar = 200 nm. (**C**) Time-dependent fold increase in fluorescence of Thioflavin T (ThT) in the presence of spike-derived peptides, compared to the fluorescence of ThT in the absence of peptides.

The spike protein segments, S192 (sequence 192-211 FVFKNIDGYFKIYSKHTPIN) and S194 (sequence 194-203 FKNIDGYFKI), were prepared and aggregated using an established protocol with slight modification^11,20^. The aggregation of S192 and S194 peptides was monitored and characterized by SDS-PAGE gel electrophoresis, transmission electron microscopy (TEM) and a Thioflavin T (ThT) fluorescence assay, as shown in Fig. 2. SDS-PAGE gel analysis revealed that non-aggregated S192 displayed a monomer band and a dimer band, whereas the aggregated S192 exhibited a smear of high molecular weight species (Fig. 2A), consistent with the formation of amyloid-like aggregates (For a full, unedited gel image, please see Fig. S1 in the Supporting Information)^20^. For both non-aggregated and aggregated S194, however, no discernible bands were observed by SDS-PAGE (despite confirming the presence of this peptide in the sample by UV absorbance), possibly due to the small size of S194 peptide^21,22^. The ThT fluorescence assay, which measures the increase in ThT fluorescence upon binding to soluble amyloids, showed that the solution of S192 peptide presented an increase in ThT fluorescence over time (Fig. 2C), which was similar to previous characterization of the aggregation kinetics of this peptide^11^ and was consistent with the formation of amyloid-like species^23^. The S194 peptide solution did not elicit a fluorescence response to ThT over the same period of time. TEM images (Fig. 2B) further confirmed the presence of fibrillar structures from aggregated S192, supporting the amyloid-forming properties of this peptide (see Fig. S2 in the Supporting Information for TEM images from S192 samples taken at 0, 3, and 24 h after incubation).

**Figure 3:**
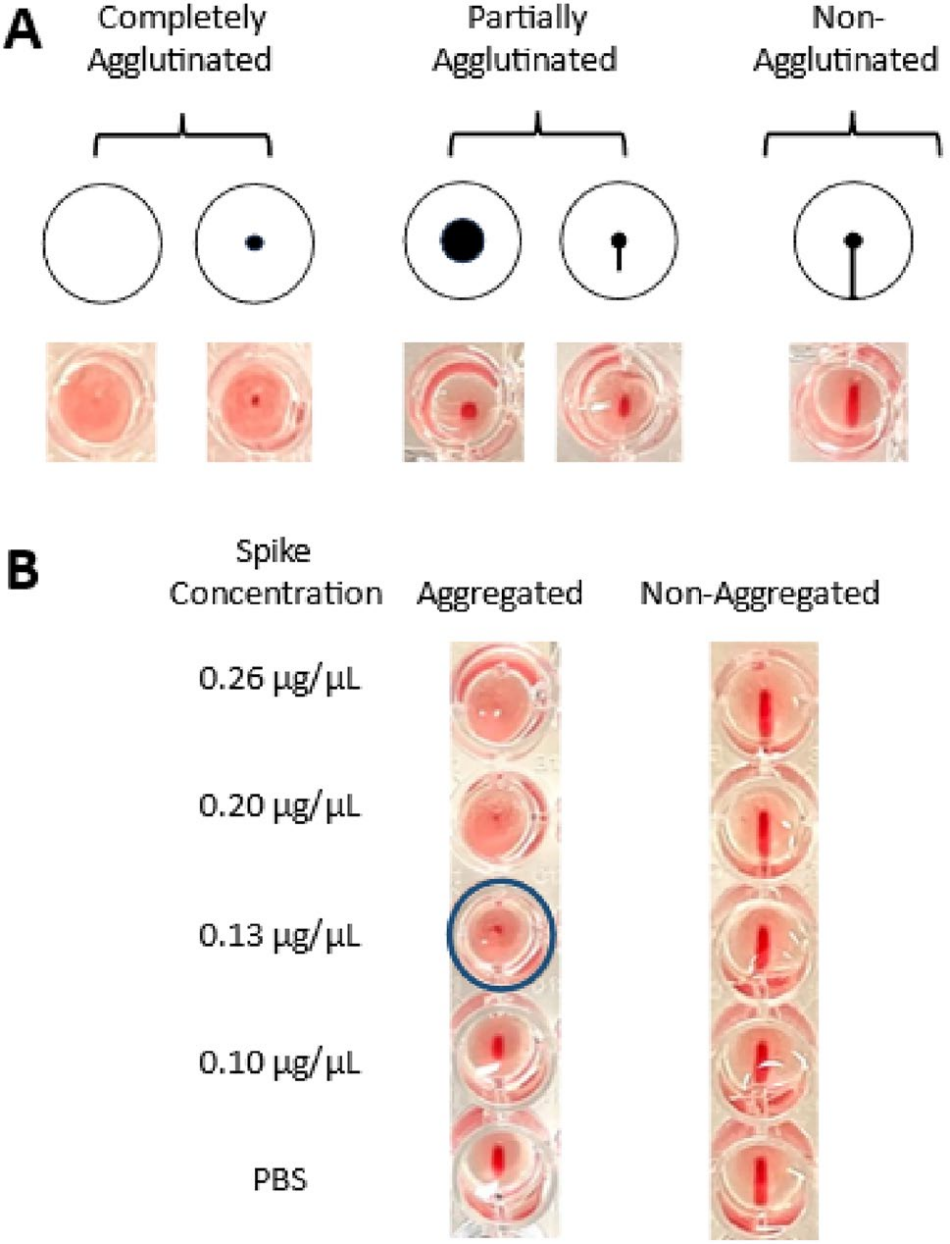
Interpretation of a Hemagglutination Assay (HA) in the presence of different concentrations of aggregated or non-aggregated S192 peptide. (**A**) Agglutination patterns of RBCs: completely agglutinated, partially agglutinated and non-agglutinated. (**B**) HA of blood in the presence of increasing concentrations of aggregated and non-aggregated S192 peptides. The blue circled well depicts the minimum concentration of aggregated S192 peptides that was found to induce complete hemagglutination under the experimental conditions.

The results of the peptide aggregation studies of S192 and S194 suggested only S192 formed amyloids in our hands. We, therefore, only moved forward with S192 to investigate whether aggregated S192 could induce agglutination of red blood cells (RBCs) using an established Hemagglutination Assay (HA)^17,24,25^. In this experiment, we incubated varying concentrations of aggregated S192 with a consistent amount of RBCs in V-shaped 96-well microtiter plates at room temperature for one hour. For comparison, non-aggregated S192 peptides were also incubated under identical conditions at the same concentrations. After incubation, the plate was tilted for at least 30 s before examining the results. Tilting the plate is crucial for distinguishing the three types of agglutination patterns: completely agglutinated, partially agglutinated, and non-agglutinated^17,25,26^. Representative photographic images of these agglutination patterns can be seen in Fig. 3A. Briefly, when RBCs are completely agglutinated, a small red spot or lattice forms at the bottom of the well. Non-agglutinated cells, on the other hand, result in the formation of a red teardrop in the well. Partially agglutinated cells appear as a circular red spot often with a halo and with a larger diameter comparing to a fully agglutinated sample, or a shortened teardrop comparing to non-agglutinated blood samples. These observations and interpretations from the HA are also reported by other groups carrying out hemagglutination studies^17,25–27^. Based on the HA experiments, aggregated S192 was found to fully agglutinate RBCs at the concentration of 0.13 μg/μL or higher, as demonstrated in Fig. 3B. In contrast, non-aggregated S192 did not induce hemagglutination at all concentrations tested up to 0.26 μg/μL. While previous studies have reported that the full-length spike protein can induce hemagglutination through interactions between N-glycans on the spike protein and host cell glycoconjugates^5,24^, to our knowledge this result represents the first evidence of the agglutination of RBCs induced by non-glycosylated amyloid-like S192 aggregates.

**Figure 4:**
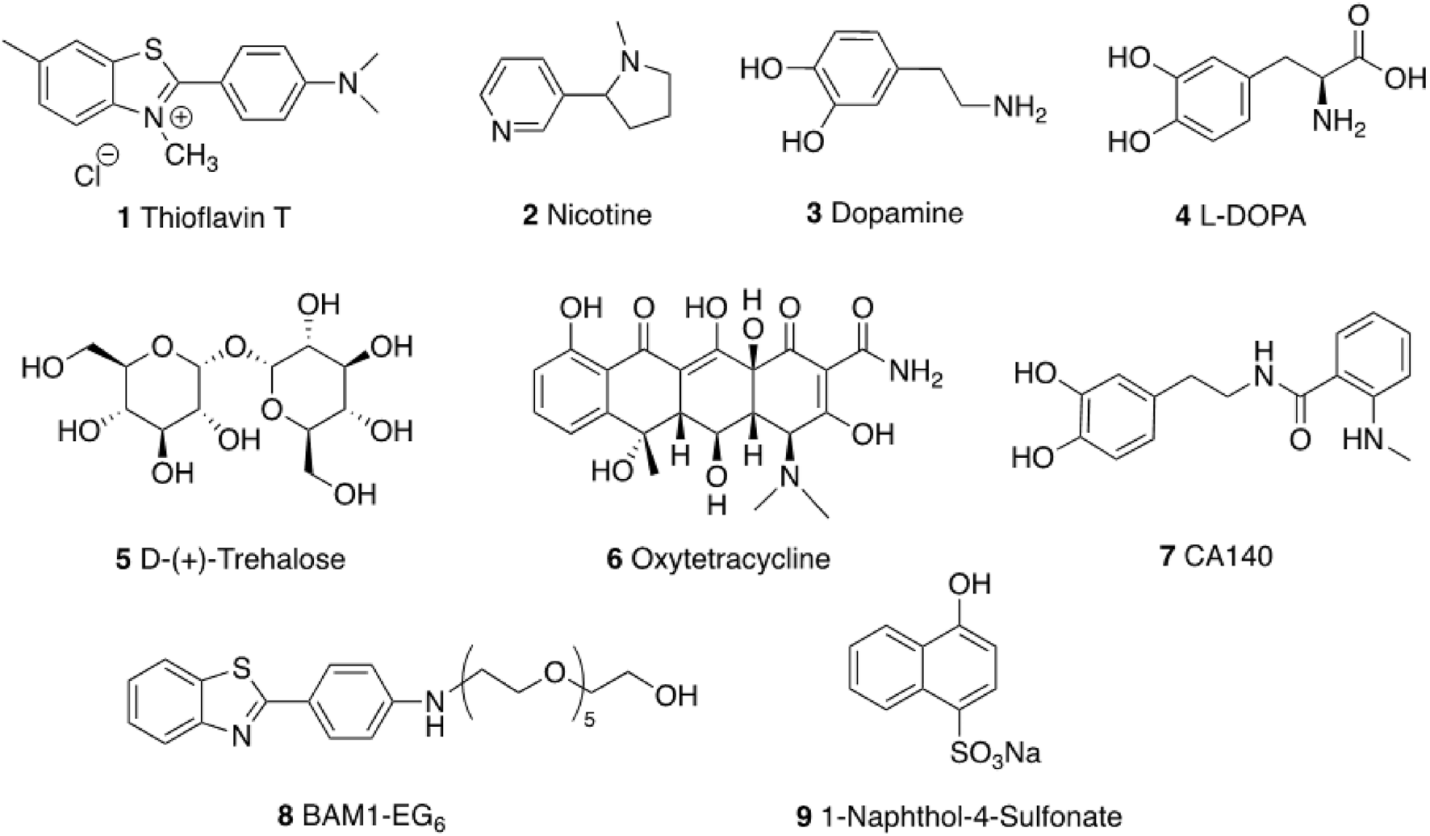
Structure of amyloid-binding molecules used in the Hemagglutination Inhibition Assay (HIA).

In order to test one possible approach to inhibiting hemagglutination induced by S192 aggregates, we next performed a Hemagglutination Inhibition Assay (HIA) in the presence of small molecules that have been reported to form bio-resistive molecular coatings on amyloids. Molecular coatings on amyloid surfaces have emerged as novel therapeutic tools with broad technological applications in diverse biomedical fields^28–32^. For instance, small molecules that form bio-resistive coatings on the surface of aggregated β-amyloid peptides have been reported to inhibit the interactions of cellular proteins with amyloid fibrils and reduce amyloid-induced oxidative stress and toxicity associated with Alzheimer’s disease^30,33,34^. Molecular coatings on amyloids have also been shown to inhibit enhancement of HIV virus infection induced by natural SEVI amyloid fibrils found in semen^28,32^. We, therefore, used a modified protocol of a previously reported HIA^25^ to screen a set of 9 molecules (Fig. 4) consisting of compounds **1–8**^30,31,34,35^ known to inhibit the interaction between amyloids and cellular proteins. We also included compound **9**^29^ in our screen as a negative control, since this molecule was reported not to bind to amyloids. We selected these molecules for this initial screen since they have good water solubility and are colorless or do not have substantial overlapping color with the visual readout of the assay.

**Figure 5:**
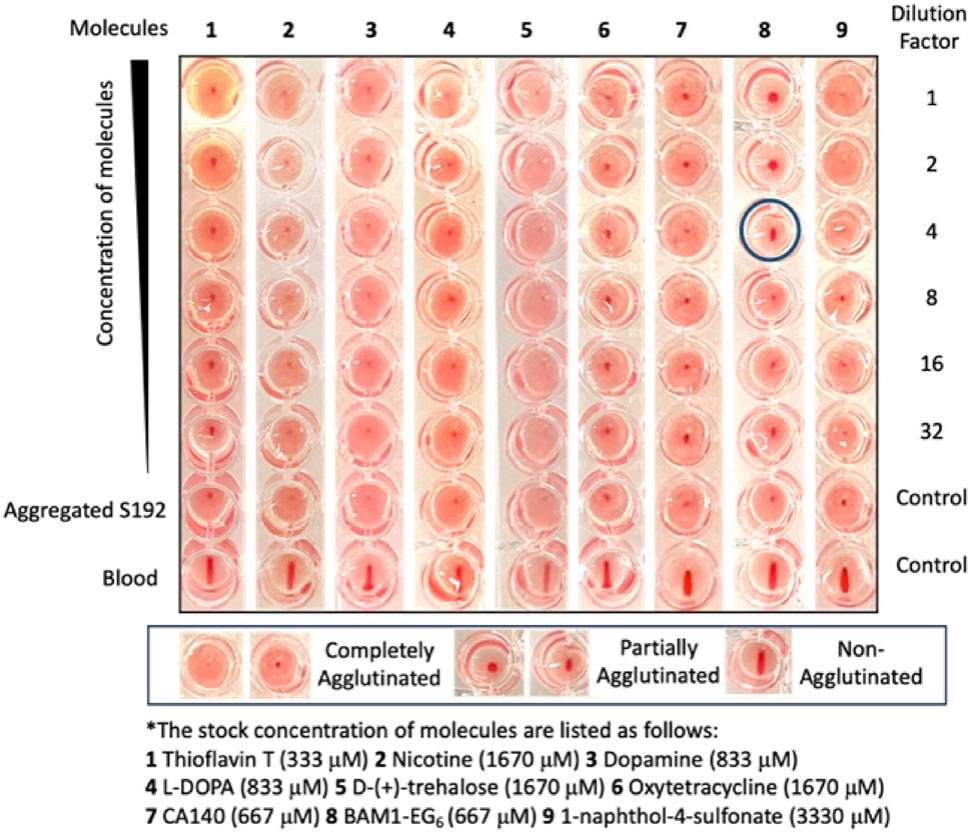
Inhibition of spike-induced hemagglutination with small molecules. The blue circled well depicts the minimum concentration of BAM1-EG_6_ that was found to partially inhibit the aggregated S192-induced hemagglutination.

To perform the Hemagglutination Inhibition Assay (HIA), a twofold series of dilutions of small molecules was carried out across the plate. A constant 0.13 μg/μL of aggregated spike S192 peptides were added to each well and incubated with the small molecules for 1 h at room temperature. RBCs were then added to the plate and allowed to settle for 1 h at room temperature quiescently. The plate was tilted for 30 s, and the results were recorded (Fig. 5). For each plate, a blood-only control without the addition of aggregated S192 was included to ensure that the blood did not self-agglutinate during the experiment. An aggregated S192 peptide-only control without any added molecules was also included to verify the integrity of the spike peptide solution. Additionally, each compound was tested with RBCs in the absence of aggregated spike peptides under the same condition to control for any observed effects of compound-induced HA.

Compound **1–7** and **9**, showed no inhibitory effects on S192-induced hemagglutination up to the limit of solubility of each compound (for ThT, we used the maximum concentration that did not interfere colorimetrically with the assay readout). Compound **8**, BAM1-EG_6_, on the other hand, was found to partially inhibit the hemagglutination, as indicated by the larger central red spot and halo pattern or shortened teardrop pattern observed in the assay (Fig. 5).

**Figure 6:**
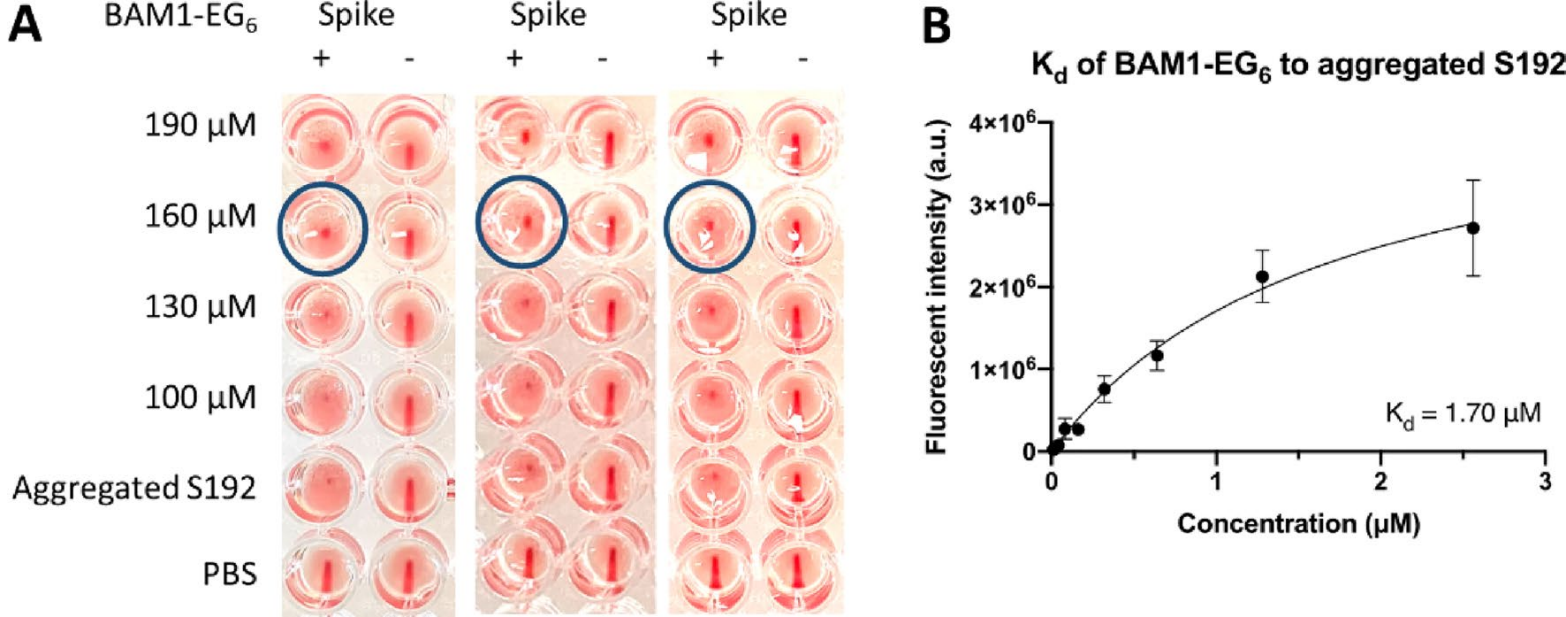
Characterization of the inhibition activity of BAM1-EG_6_ towards S192-induced hemagglutination and evidence of binding of BAM1-EG_6_ to aggregated S192. (**A**) Triplicate experiments of a HIA of aggregated S192 in the presence of BAM1-EG_6_ at 30 μM concentration steps. The blue circled wells depicts the minimum concentration of BAM1-EG_6_ that was found to partially inhibit the spike fibril induced hemagglutination. (**B**) A fluorescence versus concentration curve for BAM1-EG_6_ used to estimate its binding affinity (K_d_) to aggregated S192.

To determine the lowest effective concentration for the inhibition of hemagglutination by compound **8**, we conducted a more detailed HIA at 30 μM steps in concentration. As depicted in Fig. 6A, BAM1-EG_6_ exhibited partial inhibition of HA at a concentration of 160 μM or above. We also assessed the binding affinity (K_d_) of BAM1-EG_6_ to aggregated S192, which was determined to be 1.7 μM (Fig. 6B). For comparison, the reported binding affinity of BAM1-EG_6_ to aggregated Aβ was 270 nM^34^. While molecules **1–8** were previously shown to inhibit the binding of various cellular proteins to amyloids^29–31,34^, the concentration required for maximal inhibition and the extent of inhibition of protein-amyloid interactions observed for each molecule varied widely. These differences in inhibitory activity were attributed to potential differences in the density of binding sites for molecules on the amyloid surface, which could be highly dependent on the structure of the small molecule and the protein composition of the amyloid.

## Discussion

Overall, the present study provides evidence that the amyloidogenic peptide derived from the SARS-CoV-2 spike protein, S192, can induce the agglutination of red blood cells, which could potentially contribute to the microvascular morbidities observed in COVID-19 patients. It is noteworthy that S192 lacks any known glycosylation sites, and only the aggregated form of S192 is capable of inducing hemagglutination. This result suggests a novel mechanism for agglutination activity for this spike protein metabolite that operates independently of the interaction of the glycosylation sites with cell surface sialoglycoproteins. Furthermore, this study identifies an amyloid-binding molecule, BAM1-EG_6_, that can partially intervene in spike peptide-induced hemagglutination, which serves as proof-of-concept for a potential therapeutic strategy that involves amyloid-binding agents to attenuate vascular occlusive complications caused by the presence of metabolic products of the SARS-CoV-2 spike protein. Previous studies have shown that multivalent amyloid-targeting groups^36^ and the use of non-covalent secondary interactions between molecules^34^ can significantly increase the binding of small molecules to amyloid targets by orders of magnitude. These strategies to improve affinity, together with polymeric display of amyloid targeting molecules to increase steric bulk^32^, have also been shown to substantially increase the efficacy of biomolecular coatings to inhibit the interaction of amyloids with cellular proteins, virus particles, and cells. Current efforts are focused on employing such strategies to generate molecules with improved capability to inhibit hemagglutination induced by aggregated forms of SARS-CoV-2 spike protein metabolites.

## Methods

### Materials

Bovine blood (Cat# BBE250, 250 mL Bovine Blood stabilized with EDTA) was purchased from HemoStat Laboratories (Dixon, CA). BAM1-EG_6_ and CA140 were synthesized as previously described^34,35^. All other compounds were purchased from Sigma-Aldrich (St. Louis, MO). The V-bottomed 96-well micro test plates (Cat# 82.1583.001) were purchased from SARSTEDT (Newton, NC).

### Aggregation of peptide fragments derived from the SARS-CoV-2 spike protein

The SARS-CoV-2 spike segment S192 (sequence 192-211 FVFKNIDGYFKIYSKHTPIN) and S194 (sequence 194-203 FKNIDGYFKI) were purchased from Biopeptide Co., Inc. (San Diego) with > 98% purity characterized by HPLC. The peptides were prepared and aggregated using established protocol with slight modification^11,20^. The main difference was the rigorous removal of HFIP through lyophilization prior to initiating aggregation, as we found that HFIP can lead to false positive results in the hemagglutination assay (data not shown). Briefly, 1 mg of peptide was dissolved in 100 μL of 1, 1, 1, 3, 3, 3-hexafluoro-2-propanol (HFIP) to reach a final peptide concentration of 10 mg/mL. The samples were sonicated in a water bath for 15 min, following by a 24-h incubation period at room temperature with gentle shaking. Ice cold water was then added to the peptide solution at a 2:1 H_2_O:HFIP volume ratio, followed by an additional 2-h incubation at room temperature. The solution was then frozen at − 80 °C for a minimum of 24 h and lyophilized for 3 days. The lyophilized peptide was then stored at − 80 °C until needed. To form amyloid-like aggregates, the lyophilized peptide was dissolved in 50 μL of DMSO to reach a final concentration of 20 mg/mL, followed by the addition of phosphate buffered saline (PBS) pH 7.4 to attain a final concentration of 1 mg/mL in PBS buffer containing 5% DMSO. The peptide solution was then incubated at 37 °C with continuous shaking at 1000 rpm on an orbital shaker for 24 h.

### Thioflavin T assay

In the Thioflavin T (ThT) assay, ThT and the unaggregated spike-derived peptides were combined in a PBS buffer (pH 7.4) inside a black 96-well plate (Costar, cat# 3915). Their final concentrations were 10 μM for ThT and 0.1 mg/mL or 1 mg/mL for the peptide. Using a SpectraMax i3x (Molecular Devices, LLC.), we set the excitation wavelength at 440 nm and recorded the emission spectrum between 475 and 700 nm with a step of 1 nm at various incubation times up to 21 h. We subtracted the buffer blank values and used the fluorescence intensity of ThT at 480 nm, both in the presence and absence of peptides, to determine the fluorescence fold increase. We analyzed and visualized the data using GraphPad Prism (version 8.0.0 for macOS, GraphPad Software, San Diego, California USA).

### Transmission electron microscopy of aggregated S192 peptide

Carbon-coated copper grids (Ted Pella, Cat# 01754-F) were treated by air plasma and floated on droplets of solutions containing aggregated spike peptide S192 (20 μL, 0.1 mg/mL) for 5 min. The grids were then washed three times with double distilled water for 30 s each time and stained with 2% uranyl acetate for 1 min. The grids were blotted dry and air dried for at least 20 min before insertion into the microscope holder. Transmission electron microscopy (TEM) imaging was performed using a FEI Tecnai Spirit G2 BioTWINTEM microscope operating at 80 keV.

For monitoring the aggregation of spike peptide S192 by TEM, the peptide solution was collected at three time points: t = 0 h (0 h), t = 3 h (3 h) and t = 24 h (24 h), and diluted to 0.1 mg/mL in PBS buffer, pH 7.4. Air plasma-treated carbon-coated copper grids were floated on diluted peptide solutions for 5 min before negative staining with 2% uranyl acetate. TEM was performed on air dried grids using a JEOL 1400 plus microscope operating at 80 keV.

### Hemagglutination assay (HA)

The bovine blood sample was diluted at a 1:40 volume ratio using PBS, pH of 7.4. Specifically, 200 μL of the bovine blood was mixed with 8 mL of PBS. The solution was resuspended by inverting gently 20 times. Different concentrations of aggregated spike peptides were dispensed at 50 μL per well to a V-bottomed 96-well microtiter plate. 50 μL of the diluted blood were added to each well of the plate. 50 μL of PBS was added to maintain the same total volume (150 μL) as in the hemagglutination inhibition assay. The solution was mixed via pipette several times for each well. The plate was incubated at room temperature for 1 h quiescently, then tilted for 30 s prior to recording results. The results were recorded using the camera function on a mobile phone using the zoom function to obtain a complete field. For each sample, a control well containing no aggregated peptide was included.

### Hemagglutination inhibition assay (HIA)

The bovine blood sample was diluted at a 1:40 volume ratio using PBS, pH of 7.4. Specifically, 200 μL of the bovine blood was mixed with 8 mL of PBS. The mixture was gently inverted 20 times to ensure thorough resuspension. Different concentrations of small molecules were dispensed into the plate at 50 μL per well. The same amount of aggregated spike peptide, 20 μL of a 1 mg/mL aggregated S192 solution, was added at 50 uL per well in the plate. The solution was allowed to equilibrate for 1 h at room temperature with gentle shaking. Diluted blood was then added at 50 uL per well and allowed to settle for 1 h. After incubation, the plate was tilted for 30 s, and the results were recorded. The minimal effective concentration of molecule needed for inhibition was defined as the well with the lowest concentration of molecule in which the teardrop, indicating no agglutination, was not observed. A partial teardrop shape was regarded as a positive result.

### Measurement of the binding affinity of BAM1-EG_6_ to aggregated spike peptide S192

Binding of BAM1-EG_6_ to aggregated spike peptide S192 was measured according to a previously described assay^8^. Briefly, 200 μL of various concentrations of BAM1-EG_6_ in PBS were added to a microcentrifuge tube in the presence and absence of 10 μg of aggregated spike peptides. After overnight incubation, the solution was centrifuged at 16,000×*g* for 30 min at 4 °C. The supernatants were discarded, and the pellet was resuspended in 220 μL of fresh PBS. Fluorescence of bound and unbound molecules was determined by excitation at 355 nm and recording the intensity of emission at 420 nm. A one-site specific binding algorithm was used to determine K_d_: Y = B_max_ × X/(K_d_ + X), where X is the concentration of small molecule, Y is the specific binding fluorescence intensity, and B_max_ is the apparent maximal observable fluorescence upon binding to aggregated spike peptide.

### Supplementary Information

Supplementary Figures.

## Data Availability

All data generated or analyzed during this study are included in this published article or are available from the corresponding author on reasonable request.
