# Supplementary material for: Reduction of hemagglutination induced by a SARS-CoV-2 spike protein fragment using an amyloid-binding benzothiazole amphiphile
Source: Sci Rep. 2024 May 29;14:12317. doi: 10.1038/s41598-024-59585-4 (PMC11137076; doi:10.1038/s41598-024-59585-4)
Supplement: Supplementary file 1 — Supplementary Figures. [file 41598_2024_59585_MOESM1_ESM.docx]

**Supporting Information**

Reduction of Hemagglutination Induced by a SARS-CoV-2 Spike Protein Fragment Using an Amyloid-Binding Benzothiazole Amphiphile

Meihan Li, Sascha Castro Lingl, and Jerry Yang^*^

University of California, San Diego, Department of Chemistry and Biochemistry, 9500 Gilman Drive, La Jolla, 92093-0358, USA

**Supporting Information pages**

**Figure S1:** Silver-stained SDS-PAGE gel of SARS-CoV-2 spike segments, S192 and S194 S1

**Figure S2:** Time course transmission electron microscopy (TEM) images of S192 peptides S2

**Supporting references** S3


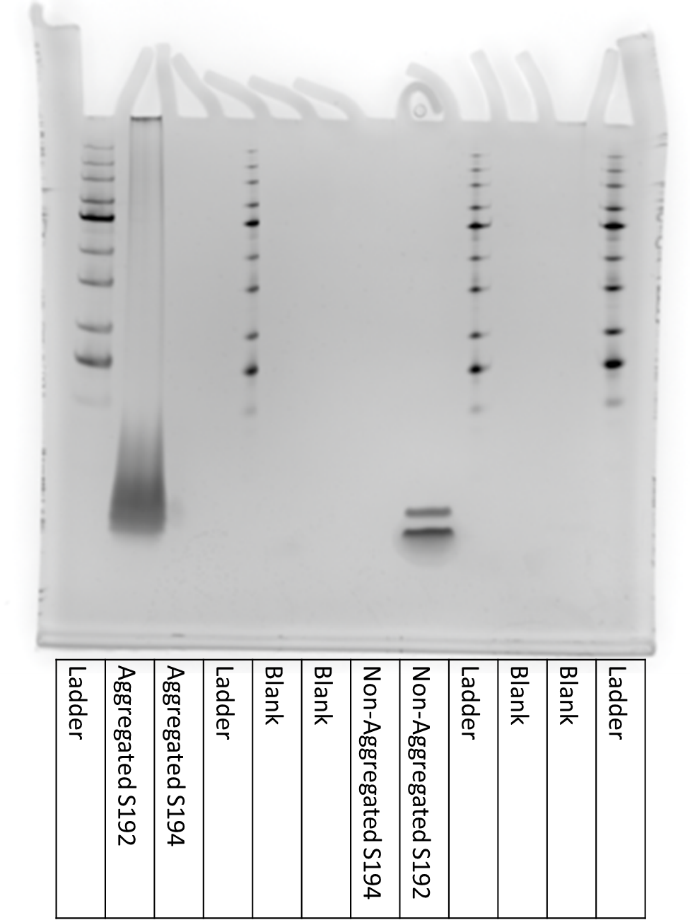


**Figure S1.** Full, unedited silver-stained SDS-PAGE gel of SARS-CoV-2 spike segments, S192 and S194.


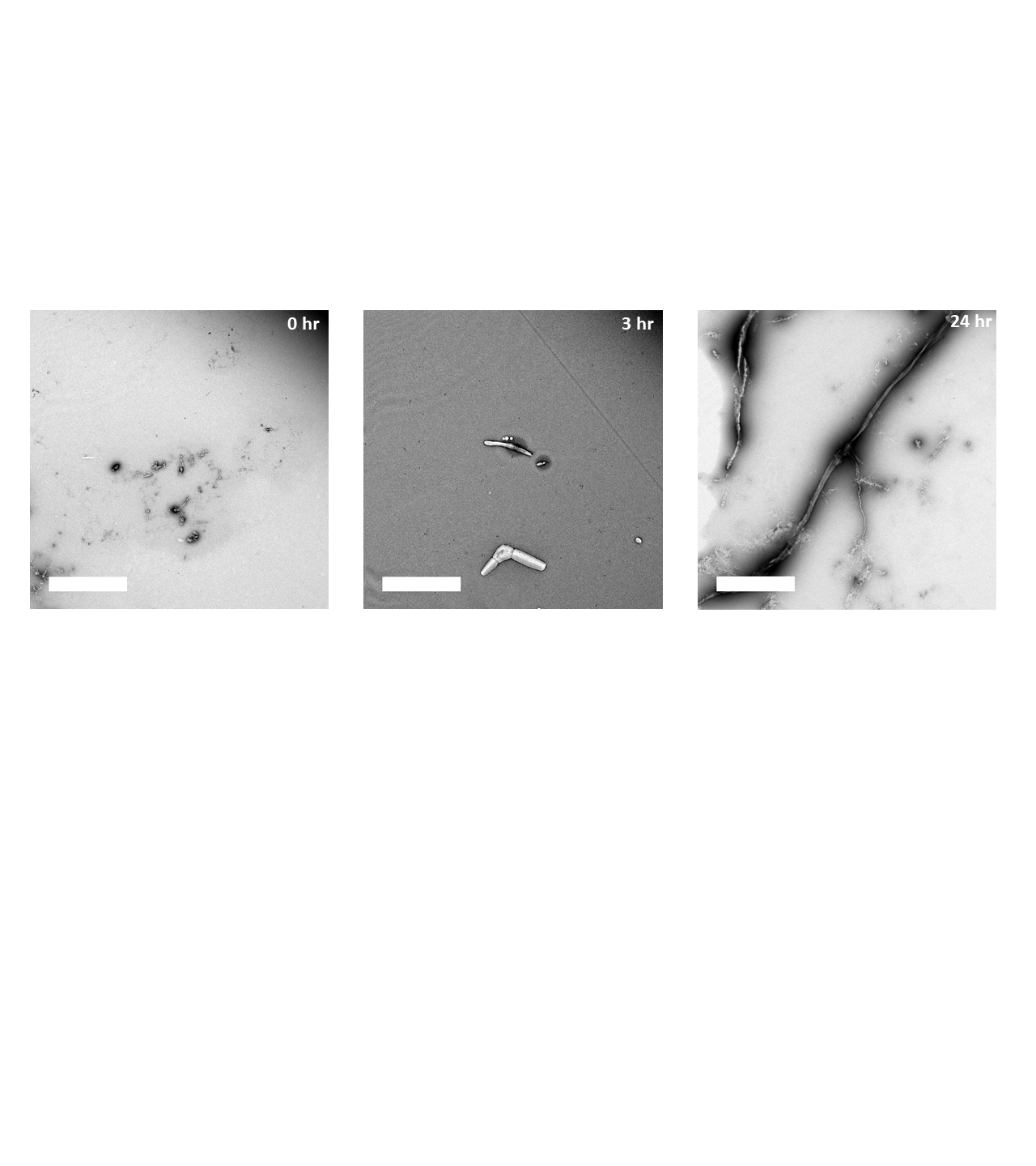


**Figure S2.** Negatively stained transmission electron microscopy (TEM) ultrastructure of S192 peptide samples at different timepoints after incubation. Peptide S192 was prepared and aggregated following established protocol with slight modification.^1,2^ The S192 peptide solution was collected and diluted to 0.1mg/mL in PBS buffer, pH 7.4 at time points: t = 0 hour (0 hr), t = 3 hours (3 hr) and t = 24 hours (24 hr). Air plasma treated carbon-coated copper grids were floated on diluted peptide solutions for 5 minutes before negative staining with 2% uranyl acetate. TEM was performed on air dried grids using a JEOL 1400 plus microscope operating at 80KeV. Scale bar = 2 μm.

**Supporting references:**

1. Nyström, S. & Hammarström, P. Amyloidogenesis of SARS-CoV-2 spike protein. *J. Am. Chem. Soc.* **144**, 8945–8950 (2022).
2. Prangkio, P., Yusko, E. C., Sept, D., Yang, J. & Mayer, M. Multivariate analyses of amyloid-beta oligomer populations indicate a connection between pore formation and cytotoxicity. *PLoS One* **7**, e47261 (2012).
